# Escalation of Germinal Center Responses in Chronic *Litomosoides sigmodontis* Filarial Infection

**DOI:** 10.1002/eji.202451400

**Published:** 2025-05-13

**Authors:** Teresa Steffen, Jesuthas Ajendra, Marianne Koschel, Alexander Palmen, Hannah Wegner, Frederic Risch, Luisa Bach, Manuel Ritter, Marc P. Hübner, Dirk Baumjohann

**Affiliations:** ^1^ Medical Clinic III for Oncology Hematology, Immuno‐Oncology and Rheumatology University Hospital Bonn University of Bonn Bonn Germany; ^2^ Institute for Medical Microbiology Immunology and Parasitology (IMMIP) University Hospital Bonn University of Bonn Bonn Germany; ^3^ German‐West African Centre for Global Health and Pandemic Prevention (G‐WAC) Partner Site Bonn Bonn Germany; ^4^ German Center for Infection Research (DZIF) Partner site Bonn‐Cologne Bonn Germany

**Keywords:** B cell, germinal center, *Litomosoides sigmodontis*, T_FH_ cell, T_FR_ cell

## Abstract

T follicular helper (T_FH_) cells are the primary CD4^+^ T helper cell subset providing help to B cells for efficient antibody responses in vaccination, allergy, autoimmunity, and infectious diseases. Despite their critical involvement in immunity, T_FH_ cells’ specific role in filarial infections remains unclear. Using the rodent filarial model *Litomosoides sigmodontis*, we examined T_FH_ and germinal center (GC) B cell responses in lung‐draining mediastinal lymph nodes (medLNs) over a 110‐day infection period in naive and infected wildtype (WT) BALB/c mice, as well as eosinophil‐deficient dblGATA mice, using flow cytometry and ELISA. We observed robust and prolonged T_FH_ and GC B cell responses in medLNs of infected BALB/c mice, along with enduring IgG1 antibody responses next to a persistent systemic humoral immune response. We further provide evidence of dysregulated T_FH_/T follicular regulatory (T_FR_) cell ratios in medLNs. Finally, elevated T_FH_ cell frequencies in medLNs of dblGATA mice reaffirm the significant role of eosinophils during chronic infection. In conclusion, our findings provide novel insights into population changes of T_FH_ and GC B cells during filarial infection.

## Graphical abstract

T follicular helper (T_FH_) cells provide essential help to B cells in germinal centers (GCs). T_FH_ and GC B cell responses are long‐lasting in draining mediastinal lymph nodes of *Litomosoides sigmodontis*‐infected BALB/c mice, a rodent model of filarial infection.

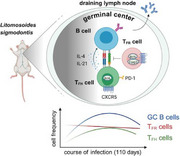

## 1 Introduction

CD4^+^ T helper cells regulate various aspects of adaptive immunity [1, 2]. While it was previously thought that Th2 cells primarily assist B cells in antibody production, it is now recognized that T_FH_ cells represent the principal CD4^+^ T cell subset specialized in this function [3, 4]. T_FH_ cells are critically involved in the establishment of germinal centers (GCs) and play an essential role in the generation of robust antibody responses against various pathogens [5]. T_FH_ cells are characterized by the expression of the chemokine receptor CXCR5, the transcription factor Bcl6, as well as costimulatory and inhibitory molecules such as ICOS and PD‐1 [4]. Further, T_FH_ cells secrete various cytokines, including IL‐4 and IL‐21, which act on B cells [5]. T_FH_ cells themselves can express transcription factors normally associated with other distinct CD4^+^ T cell subsets [4]. For example, T follicular regulatory (T_FR_) cells share characteristics of Bcl6 and CXCR5‐expressing T_FH_ cells as well as Foxp3‐expressing T regulatory (T_REG_) cells [4].

Filariae cause neglected tropical diseases such as onchocerciasis and lymphatic filariasis [6] and elicit strong type 2‐driven immune responses involving canonical Th2 cell responses, ILC2s [7], and eosinophils [6, 8, 9]. Further, type 2 cytokine production, involving IL‐4 and IL‐10, and high IgG4 and IgE antibody levels are hallmarks of human filarial infection [10]. *Litomosoides sigmodontis* is a rodent model of filariasis mirroring human filarial infections and thus facilitates the investigation of anti‐filarial immunity in vivo [11]. Susceptible BALB/c mice enable the complete life cycle of *L. sigmodontis*, with 50% of animals developing microfilaremia [11]. Wildtype (WT) C57BL/6 mice are regarded as semisusceptible as they clear the infection shortly after the molt into adult worms and do not present with microfilaremia [9, 12]. In contrast, T cell and B cell‐deficient *Rag2^–/–^* C57BL/6 mice are susceptible to filarial infections and develop microfilaremia, signifying a critical role of T and B cells for the elimination of infection [13].

Since antibody responses are driven by T_FH_ cells in GCs in secondary lymphoid organs (SLOs) [5], this implicates T_FH_ cells as critical constituents of the antifilarial humoral immune response. Further, host CD4^+^ T cell responses are associated with disease manifestations in human filarial infections, such as in lymphatic filariasis [10]. Surprisingly, however, the role of T_FH_ and GC B cells in infections within the rodent model *L. sigmodontis* is largely unknown [11, 14]. To fill this gap, we analyzed T_FH_ and GC B cell responses of infected BALB/c mice during the parasitic life cycle of *L. sigmodontis* and found that immune responses in medLNs remained persistent throughout infection and that medLNs exhibited dysregulated T_FH_/T_FR_ cell ratios. Additionally, we observed enduring local IgG1^+^ GC B cell responses, validated systemic humoral immune responses, and found that T_FH_ cell frequencies in medLNs were significantly higher in eosinophil‐deficient dblGATA mice.

## 2 Results and Discussion

### 2.1 Chronic T_FH_ Cell Responses as Potential Hallmark of the Adaptive Anti‐Filarial Immune Response to L. sigmodontis infection

**FIGURE 1:**
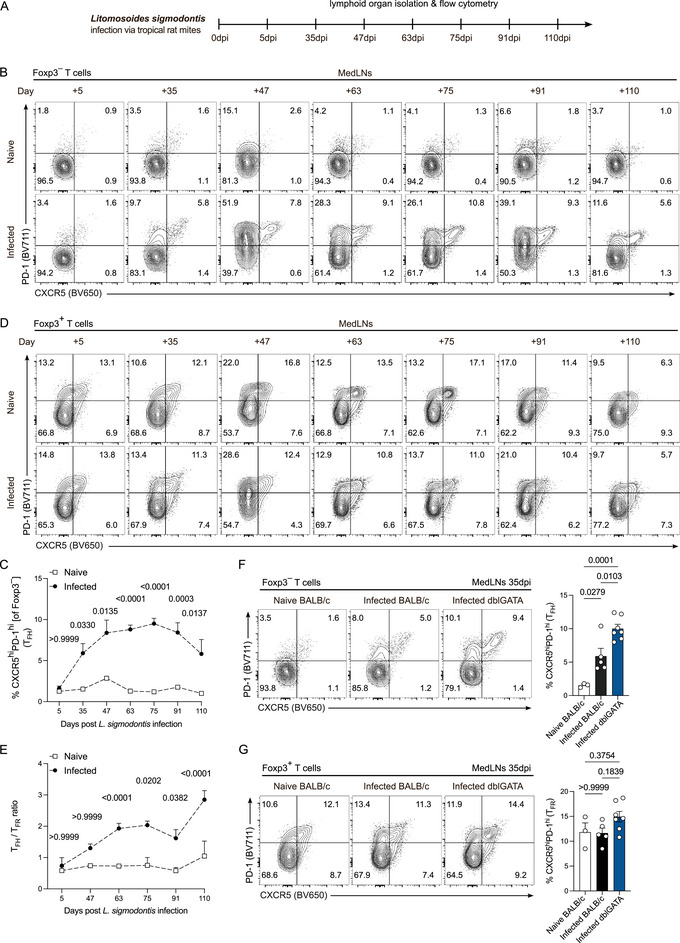
Strong and prolonged T_FH_ cell responses and dysregulated T_FH_/T_FR_ cell ratios in medLNs during *L. sigmodontis* infection. (A) Experimental study design. WT BALB/c mice were infected with *L. sigmodontis* and analyzed at different time points postinfection. (B) Representative flow cytometry contour plots show the expression of CXCR5 and PD‐1 by live CD4^+^CD8^–^CD19^–^CD44^+^Foxp3^–^ T cells in medLNs of naive (top) and infected (bottom) WT BALB/c mice on 5, 35, 47, 63, 75, 91, and 110 dpi. Naive BALB/c controls remained uninfected. (C) Quantification of CXCR5^hi^PD‐1^hi^ T_FH_ cell frequencies in medLNs of naive and infected BALB/c mice. (D) Representative flow cytometry contour plots show the expression of CXCR5 and PD‐1 by live CD4^+^CD8^–^CD19^–^Foxp3^+^ T cells IN medLNs of naive (top) and infected (bottom) BALB/c mice. (E) Quantification of T_FH_/T_FR_ cell ratios during infection in medLNs of naive and infected BALB/c mice. Ratios were calculated using T_FH_ and T_FR_ cell counts. (F) Representative flow cytometry contour plots show the expression of CXCR5 and PD‐1 by live CD4^+^CD8^–^CD19^–^CD44^+^Foxp3^–^ T cells in medLNs of naive BALB/c, infected BALB/c, and infected dblGATA mice at 35 dpi. Quantification is shown on the right. (G) Representative flow cytometry contour plots show the expression of CXCR5 and PD‐1 by live CD4^+^CD8^–^CD19^–^Foxp3^+^ T cells in medLNs of naive BALB/c, infected BALB/c, and infected dblGATA mice at 35 dpi. Quantification is shown on the right. Time points in (B–E) represent *n* = 3–9 naive and *n* = 5–14 infected BALB/c mice. Data from 5, 35, 47, 75, 91, and 110 dpi are from one experiment. Data from 63 dpi consists of two independent experiments. Data in (F) and (G) represent *n* = 3 naive BALB/c, *n* = 5 infected BALB/c, and *n* = 7 dblGATA mice and are from one experiment. Data were analyzed with either a two‐way ANOVA with Bonferroni post hoc test comparing naive and infected group differences per timepoint (C, E) or one‐way ANOVA with Bonferroni post hoc test (F, G). Data are shown as the mean ± SEM. *p*‐Values are displayed as numerical values above the corresponding data points in the graphs.

In order to track T cell population changes in *L. sigmodontis* infection, a longitudinal study was performed over the course of 110 days post infection (dpi). Given that adult worms reside in the pleural cavity and microfilariae (MF) enter the peripheral blood causing lung tissue inflammation [15], BALB/c mice were infected with third‐stage larvae over the natural route of infection and medLNs were analyzed from 5 dpi, spanning the period of postlarval molt into adult worms (28–30 dpi), through the peak of microfilaremia (70–80 dpi), and encompassing granuloma formation and worm clearance (>90 dpi) [11, 12] (Figure 1A). Infected BALB/c mice showed a significant increase in CXCR5^hi^PD‐1^hi^ T_FH_ cells in medLNs over the course of infection compared with the naive cohort, particularly after adult worms developed in the pleural cavity (after 35 dpi) (Figure 1B,C; Figures S1A and S2A). T_FH_ cell frequencies remained elevated until 110 dpi despite successful worm clearance (Figure 1C; Figure S3A), which reflects the continuous immune response during chronic infection [16]. Importantly, the elevated T_FH_ cell frequencies were only present in medLNs of infected BALB/c mice compared with frequencies found in nondraining inguinal LNs (ingLNs) at 63 dpi (Figure S2B). This implied a locally restricted T_FH_ cell response during infection. Interestingly, correlation analysis of CXCR5^hi^PD‐1^hi^ T_FH_ cell frequencies to worm burden showed significant positive correlations with each female worm counts, male worm counts, as well as both female and male worm counts (Figure S3A), while no correlation was observed between T_FH_ cell frequencies and MF counts or MF positive and MF negative animals (Figure S3B). As it is known that T_FH_ cell responses are mandated by the amount of available antigen [17], T_FH_ cell responses may be driven by parasite antigen and not MF in lung proximity.

### 2.2 *L. sigmodontis* Infection Leads to a Dysregulated T_FH_/T_FR_ Cell Ratio

MedLNs of infected BALB/c mice comprised stable CXCR5^hi^PD‐1^hi^ T_FR_ cell frequencies (of Foxp3^+^ CD4^+^ T cells) and cell counts throughout infection (Figure 1D; Figure S2C,D). Further, T_FR_ cell frequencies in ingLNs of infected mice at 63 dpi were comparable to those of naive mice (Figure S2E). However, the T_FH_ / T_FR_ cell ratios in medLNs were significantly increased in the infected cohort after 47 dpi (Figure 1E), which can also indicate an enhanced GC response [18]. T_FR_ cell frequencies did not correlate with the magnitude of the T_FH_ cell response (Figure S2F). Further, T_FR_ cell frequencies did not correlate to female worm counts, nor male or total worm counts (Figure S3C). Additionally, no correlation was observed between T_FR_ cell frequencies and MF counts/MF positivity (Figure S3B).

### 2.3 Increased T_FH_ Cell Frequencies in *L. sigmodontis*‐Infected Eosinophil‐Deficient Mice

Eosinophils contribute profoundly to the anti‐filarial immune response [6, 16, 19]. With eosinophilia as a hallmark of filarial infections, these innate cells possess multiple effector functions, such as the release of toxic granules upon recognizing opsonized filariae via Fc receptors and the release of cytokines (e.g., IL‐4) to promote Th2 immune responses [19]. Due to this functional overlap with T_FH_ cells, we investigated the potential connection between eosinophils and their help and necessity for GC responses by analyzing T_FH_ and T_FR_ cell frequencies in medLNs of infected eosinophil‐deficient dblGATA mice at 35 dpi. T_FH_ cell frequencies were significantly increased in dblGATA mice compared with naive BALB/c, and frequencies also significantly exceeded those of the infected BALB/c cohort (Figure 1F). T_FR_ cell frequencies in infected dblGATA mice were comparable to those of the naive and infected BALB/c group (Figure 1G). Our findings imply that T_FH_ cells in medLNs either compensate for the lack of eosinophils during the anti‐filarial response or eosinophils potentially impede T_FH_ cell responses, linking crucial innate to adaptive immune responses in *L. sigmodontis* infection. Our observations are also supported by the fact that dblGATA mice are more susceptible to the filariae than WT BALB/c mice, accelerating the available antigen amount, as dblGATA mice develop microfilaremia in the majority of cases while WT BALB/c mice only present with microfilaremia half of the time [16].

### 2.4 *L. sigmodontis* Infection Elicits Robust and Long‐Lasting GC B Cell Responses

**FIGURE 2:**
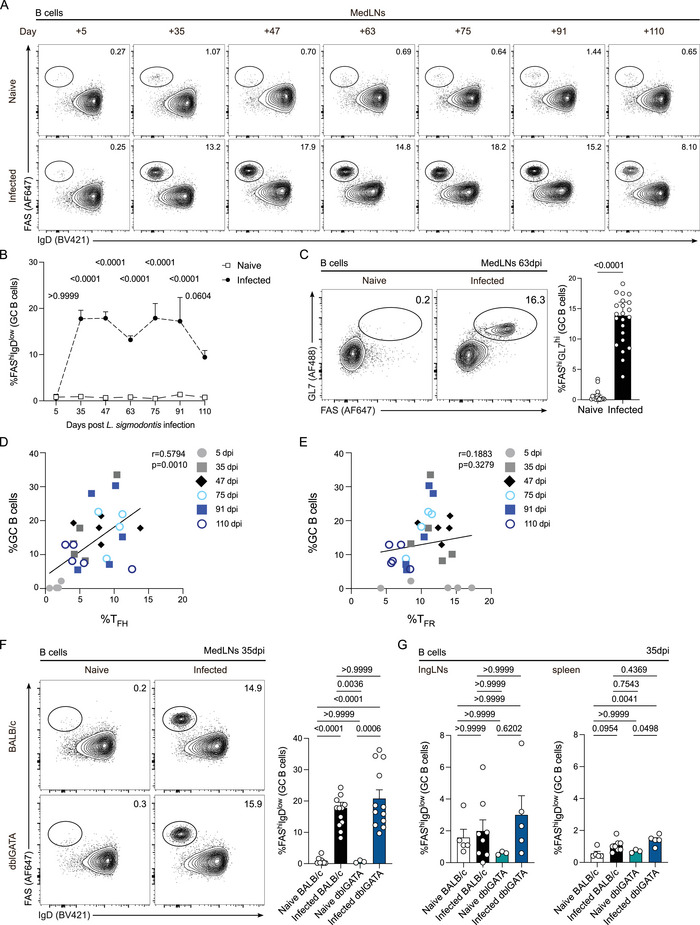
Escalation of GC B cell responses in *L. sigmodontis* infection. (A) Representative flow cytometry contour plots show the expression of FAS and IgD by live CD19^+^CD4^–^CD8^–^B220^+^ B cells in medLNs of naive (top) and infected (bottom) BALB/c mice on 5, 35, 47, 63, 75, 91, and 110 dpi. Naive BALB/c controls remained uninfected. (B) Quantification of FAS^hi^IgD^low^ GC B cell frequencies in medLNs of naive and infected BALB/c mice. (C) Representative flow cytometry contour plots show the expression of FAS and GL7 by live CD19^+^CD4^–^CD8^–^B220^+^ B cells in medLNs of naive and infected BALB/c mice at 63 dpi. Quantification is shown on the right. (D) Correlation of T_FH_ cell and GC B cell frequencies in medLNss over the course of infection. (E) Correlation of T_FR_ cell and GC B cell frequencies in medLNs over the course of infection. (F) Representative flow cytometry contour plots show the expression of FAS and IgD of live CD19^+^CD4^–^CD8^–^B220^+^ B cells in medLNs of naive BALB/c, infected BALB/c, naïve dblGATA and infected dblGATA mice at 35 dpi. Quantification is shown on the right. (G) Quantification of FAS^hi^IgD^low^ GC B cells of live CD19^+^CD4^–^CD8^–^B220^+^ B cells in ingLNs (left) and spleens (right) of naive BALB/c, infected BALB/c, naïve dblGATA, and infected dblGATA mice at 35 dpi. Time points in (A–E) represent *n* = 3–19 naive and *n* = 5–24 infected BALB/c mice. Data from 5, 47, 75, 91, and 110 dpi are from one experiment. Data from 35 and 63 dpi consist of 2 and 4 independent experiments, respectively. Data in (F) and (G) represent *n* = 3–8 naïve and *n* = 5–13 infected BALB/c and dblGATA mice. Data were pooled from 1 to 2 independent experiments. Data were analyzed with either a two‐way ANOVA with Bonferroni post hoc test comparing naive and infected group differences per timepoint (B), *t*‐test (C), or one‐way ANOVA with Bonferroni post hoc test (F, G). Correlation analysis (D, E) was performed using Pearson's correlation. Correlation coefficients are stated in the plots. Data are shown as the mean ± SEM. *p* **‐**Values are displayed as numerical values above the corresponding data points in the graphs.

Frequencies of FAS^hi^IgD^low^ GC B cells in medLNs of infected BALB/c mice were elevated over the course of infection and initially peaked between 35–47 dpi when adult worms reside in the pleural cavity (Figure 2A,B; Figure S1B, S2G). GC B cell frequencies remained elevated until at least 110 dpi, insinuating persistent GC formation even at times after worm clearance [12, 16] (Figure S3D). FAS^hi^GL7^hi^ GC B cells showed the same trend (Figure 2C). We found significant differences in GC B cell frequencies in ingLNs (Figure S2H) and spleens (Figure S2I) of naive and infected BALB/c mice, suggesting that *L. sigmodontis* infection can cause systemic GC formation at 63 dpi. However, the elevated GC B cell frequencies at 63 dpi were more prominent in medLNs (around 15%) than in ingLNs (around 2%) and spleen (around 4%). Similar to T_FH_ cells, the correlation of GC B cells to worm burden demonstrated a significant positive correlation with female worm counts, male worm counts, and total worm counts (Figure S3D), and no correlation with MF counts was observed (Figure S3B). GC B cell frequencies significantly correlated with T_FH_ cell frequencies in medLNs throughout infection (Figure 2D), as highlighted in previous research [17]; however, they did not significantly correlate with T_FR_ cells (Figure 2E). GC B cell frequencies were significantly elevated in medLNs of the infected BALB/c group and the infected dblGATA cohort compared with each respective naive group (Figure 2F). In other SLOs, we found significant differences in GC B cell frequencies in spleens of infected dblGATA mice compared with naïve mice, however intriguingly not in ingLNs (Figure 2G). Elevated B‐cell frequencies within the *L. sigmodontis* model have been found in previous studies, stating a distinct regulatory role of IL‐21 in filarial infections, as IL‐21R blockade caused elevated GC B cell frequencies in thoracic LNs as well as parasite antigen‐specific IgG1 levels [20]. Interestingly, IL‐21R blockade had no effect on T_FH_ cell expansion [20]. Overall, the dynamic B cell population changes that we observed in medLNs, which ultimately shape GCs, reflect the evolving host immune response to the parasite. Nevertheless, more research needs to be performed to elucidate the different dynamics of T_FH_ and GC B cells in dblGATA mice, as T_FH_ cells increased significantly in an eosinophil‐deficient environment compared with infected WT BALB/c mice (Figure 1F), while GC B cells did not show the same trend (Figure 2F).

### 2.5 Sustained Antibody Responses in *L. sigmodontis* Infection

**FIGURE 3:**
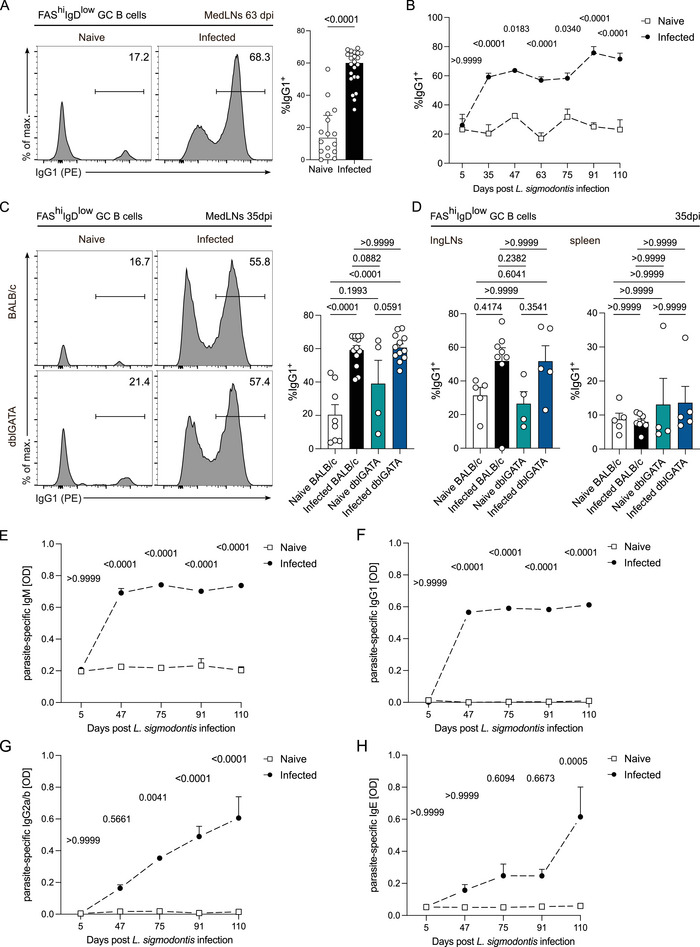
Sustained antibody responses in *L. sigmodontis* infection. (A) Representative histograms show the expression of IgG1 on live CD19^+^CD8^–^CD4^–^B220^+^FAS^hi^IgD^low^ GC B cells in medLNs of naive and infected BALB/c mice at 63 dpi. Quantification is shown on the right. (B) Quantification of IgG1^+^ GC B cell frequencies in medLNs of naive and infected BALB/c mice. (C) Representative histograms show the expression of IgG1 on live CD19^+^CD4^–^CD8^–^B220^+^FAS^hi^IgD^low^ GC B cells in medLNs of naive BALB/c, infected BALB/c, naïve dblGATA, and infected dblGATA mice at 35 dpi. Quantification is shown on the right. (D) Quantification of IgG1^+^ GC B cells in ingLNs (left) and spleens (right) of naive BALB/c, infected BALB/c, naïve dblGATA, and infected dblGATA mice at 35 dpi. (E) Parasite antigen‐specific IgM optical densities (OD) of sera from naive and infected BALB/c mice at 5, 47, 75, 91, and 110 dpi. (F) Quantification of parasite antigen‐specific IgG1. (G) Quantification of parasite antigen‐specific IgG2a/b. (H) Quantification of parasite antigen‐specific IgE. Time points in (A) and (B) represent *n* = 3–19 naive and *n* = 5–24 infected BALB/c mice. Data from 5, 47, 75, 91, and 110 dpi are from one experiment. Data from 35 and 63 dpi consist of 2 and 4 independent experiments, respectively. Data in (C) and (D) represent *n* = 4–8 naïve and *n* = 5–13 infected BALB/c and dblGATA mice. Data were pooled from 1 to 2 independent experiments. Data were analyzed with either a *t*‐test (A), two‐way ANOVA with Bonferroni post hoc test comparing naive and infected group differences per timepoint (B, E, F, G, H), or one‐way ANOVA with Bonferroni post hoc test (C, D). Data are shown as the mean ± SEM. *p*‐values are displayed as numerical values above the corresponding data points in the graphs.

More than half of the GC B cells in medLNs of infected WT BALB/c mice were IgG1^+^ after 35 dpi (Figure 3A), which persisted until at least 110 dpi (Figure 3B). This aligns with our findings of increased GC B cell frequencies (Fig. 2A, 2B, S2G). IgG1^+^ GC B cell frequencies were also significantly elevated in ingLNs (Fig. S2J) but not in the spleens of infected BALB/c mice at 63 dpi (Fig. S2K). Infected dblGATA exhibited a nonsignificant trend toward increased IgG1^+^ GC B cell frequencies compared with naive dblGATA mice (Figure 3C), while we found no significant difference between infected BALB/c and infected dblGATA. No alterations were found in ingLNs or spleens at 35 dpi (Figure 3D). Parasite antigen‐specific antibody levels in peripheral blood increased over the course of infection (Figure 3E–H). IgM (Figure 3E) and IgG1 (Figure 3F) antibody levels increased at timepoints when adult *L. sigmodontis* worms resided in the pleural cavity; levels remained elevated and stable after 47 dpi, indicative of the initial humoral immune response [12]. A continuous increase in IgG2a/b antibody levels was observed over the course of the infection (Figure 3G), indicating a shift in the quality of the antibody response. Further, parasite antigen‐specific serum IgE levels peaked at 110 dpi (Figure 3H), indicating a long‐lasting presence of parasite antigen in chronic *L. sigmodontis* infection [12].

### 2.6 Concluding Remarks

We shed new light on T_FH_ and GC B cells as potentially critical cell populations in *L. sigmodontis* infection as their population changes indicate long‐term persisting GCs throughout and beyond infection. Our study further expands our knowledge of the imbalanced immune response in chronic infection, as we found dysregulated T_FH_/T_FR_ cell ratios in medLNs. Increased T_FH_ cell frequencies in lung‐draining lymph nodes may compensate for the eosinophil‐deficient immune environment in dblGATA mice, connecting hallmark innate immune mechanisms to adaptive responses in *L. sigmodontis* infection. Interestingly, chronic *L. sigmodontis* infection has been shown to interfere with vaccination to unrelated antigens [21], which reflects the modulation capacity of filariae for self‐protection toward host–immune responses as they can generate profilarial, immunotolerant immune environments [6, 12]. Future studies should aim at further deciphering the contributions of T_FH_ and GC B cells to the escalating GCs in chronic *L. sigmodontis* infection.

## 3 Materials and Methods

### 3.1 Mice and Ethical Statement

WT BALB/c were purchased from Janvier Labs. dblGATA mice [22] were bred in the animal facilities of the Institute for Medical Microbiology, Immunology, and Parasitology (IMMIP). All animals were kept in individually ventilated cages at the IMMIP, checked daily for wellbeing, and weighed once a week during experiments to further monitor health conditions. All animal experiments were performed in accordance with European regulation and the federal law of Germany and were approved by the Landesamt für Natur‐, Umwelt und Verbraucherschutz NRW (AZ 81‐02.04.2018.A341; 81‐02.04.2020.A244; 81‐02.04.2020.A103; 02.04.40.2022.VG029).

### 3.2 Infection Model and Isolation of Worms

All experiments were performed with 7–12‐week‐old female BALB/c and dblGATA mice. Mice were naturally infected with *L. sigmodontis* by exposure and bite of *L. sigmodontis*‐infected tropical rat mites (*Ornithonyssus bacoti*). Cohorts consisted of a minimum of three naive animals and a minimum of five *L. sigmodontis*‐infected animals. Animals were euthanized with an overdose of isoflurane. Adult worm counts were determined by performing pleural cavity washes using PBS. The solution was filtered through 70 µm filters to isolate filariae. Worms were counted manually, and the sex was determined according to morphological structures under a microscope.

### 3.3 Tissue Processing and Flow Cytometry

For the isolation of mediastinal lymph nodes (medLNs), a transversal cutaneous incision was made, and the peritoneum as well as the thoracic cavity was opened. In order to assure consistency throughout the study, a total of three medLNs were isolated: two medLNs left lateral of the thymus (above the blood vessel) and one medLN right lateral to the thymus (under the right side of the heart). As controls, spleens and ingLNs were isolated. All organs were minced in dissociation buffer (2% FCS, 2 mM EDTA in PBS) between the frosted ends of two microscope slides. The cell suspension was filtered through a 70 µm filter. Red blood cell lysis was performed on spleens using 2 mL RBC lysis buffer (BioLegend). Cell suspensions were spun down at 400 g and 4°C for 4 min. Resuspended cells were counted with a BioRad TC20 automated cell counter and transferred into 96‐well V‐bottom plates for flow cytometry staining as previously described [23, 24]. Cells were washed twice with PBS. Live‐dead staining was performed using the LIVE/DEAD Fixable Near IR (780) Viability Kit (ThermoFisher Scientific). Cells were washed twice with flow cytometry staining buffer (2% FCS, 2 mM EDTA, 0.01% NaN_3_ in PBS) and then resuspended in 20 µL/well Fc‐blocking buffer (1:100 Fc block, 1:50 normal rat serum (NRS) in PBS). Next, 20 µl/well of a 2x master mix solution containing antibodies (Table S1) were added and incubated at 4°C in the dark for 30 min. Cells were washed twice, resuspended in 40 µL/well streptavidin solution, and incubated at 4°C in the dark for 20 min. For B cell staining, cells were washed twice and fixed in 100 µL/well 4% PFA in PBS for 7 min at RT in the dark before acquisition. For T cell staining, cells were fixed, permeabilized, and stained intracellularly using the Foxp3/transcription factor staining buffer set (eBioscience). 20 µL/well Fc blocking buffer in permeabilization buffer (1:100 Fc block, 1:50 NRS) were added, followed by 20 µL/well of the 2× antibody master mix. Cells were incubated for 45 min at 4°C in the dark. Samples were acquired on a BD LSRFortessa. Data were compensated with OneComp compensation beads (ThermoFisher) and analyzed with FlowJo software. Gating strategies are described in Figure S1.

### 3.4 ELISA

Serum was collected by puncture of the vena facialis. ELISA plates (Nunc) were coated with 50 µL/well of 10 µg/mL *L. sigmodontis* antigen (LsAg) diluted in PBS (pH 9.6). Plates were incubated overnight at 4°C. Nonbound LsAg was washed off the plates the next day using PBS five times. Unspecific antibody binding was blocked using 150 µL/well blocking solution (PBS + 1% BSA) and incubated for 2 h at RT. The blocking solution was washed off using PBS, plates were washed five times with PBS, and 50 µL/well of diluted serum sample was applied. All serum samples were diluted 1:100 in PBS + 1% BSA. Samples were incubated overnight at 4°C and then washed off using PBS five times. 50 µL/well of anti‐isotype (IgM, IgE, IgG1, IgG2a/b) biotin‐antibody solution diluted 1:250 in PBS + 1% BSA was added to the plates and incubated for 90 min at RT. Antibodies were washed off five times using PBS, and 50 µL/well of streptavidin‐labeled horseradish peroxidase (HRP) detection antibody solution was applied (1:250 diluted in PBS + 1% BSA) and incubated for 45 min at RT. HRP solution was washed off the plates with PBS five times. 100 µL/well TMB solution (ThermoFisher) was added and incubated at RT for approx. 15 min. The reaction was stopped using 100 µL/well H_2_SO_4_, and ODs were measured at 570 and 450 nm with a SpectraMax 190 Microplate Reader. Each sample was measured in triplicates.

### 3.5 Statistics

Statistical analyses were performed using GraphPad Prism 10 (GraphPad Software). The normality distribution of all data sets was determined by the Shapiro–Wilk test. Statistical tests are specified in each figure legend. A *p*‐value of <0.05 was considered to be statistically significant.

## Author Contributions

Teresa Steffen designed and performed most of the experiments, analyzed and interpreted the data, and wrote the manuscript. Jesuthas Ajendra, Marianne Koschel, Alexander Palmen, Hannah Wegner, Frederic Risch, and Luisa Bach performed and analyzed some experiments. Manuel Ritter and Marc P. Hübner coordinated the mouse work, provided essential tools and techniques, and contributed to the writing of the manuscript. Dirk Baumjohann designed experiments, analyzed and interpreted the data, wrote the manuscript, and provided the overall direction of the study. All authors read and approved the manuscript.

## Conflicts of Interest

The authors declare no conflicts of interest.

## Supporting information



Supplementary Materials

## Data Availability

The data that support the findings of this study are available from the corresponding authors upon reasonable request.
