# Supplementary material for: Escalation of Germinal Center Responses in Chronic Litomosoides sigmodontis Filarial Infection
Source: Eur J Immunol. 2025 May 13;55(5):e202451400. doi: 10.1002/eji.202451400 (PMC12074559; doi:10.1002/eji.202451400)
Supplement: Supplementary file 1 — Supplementary Materials [file EJI-55-e202451400-s001.pdf]

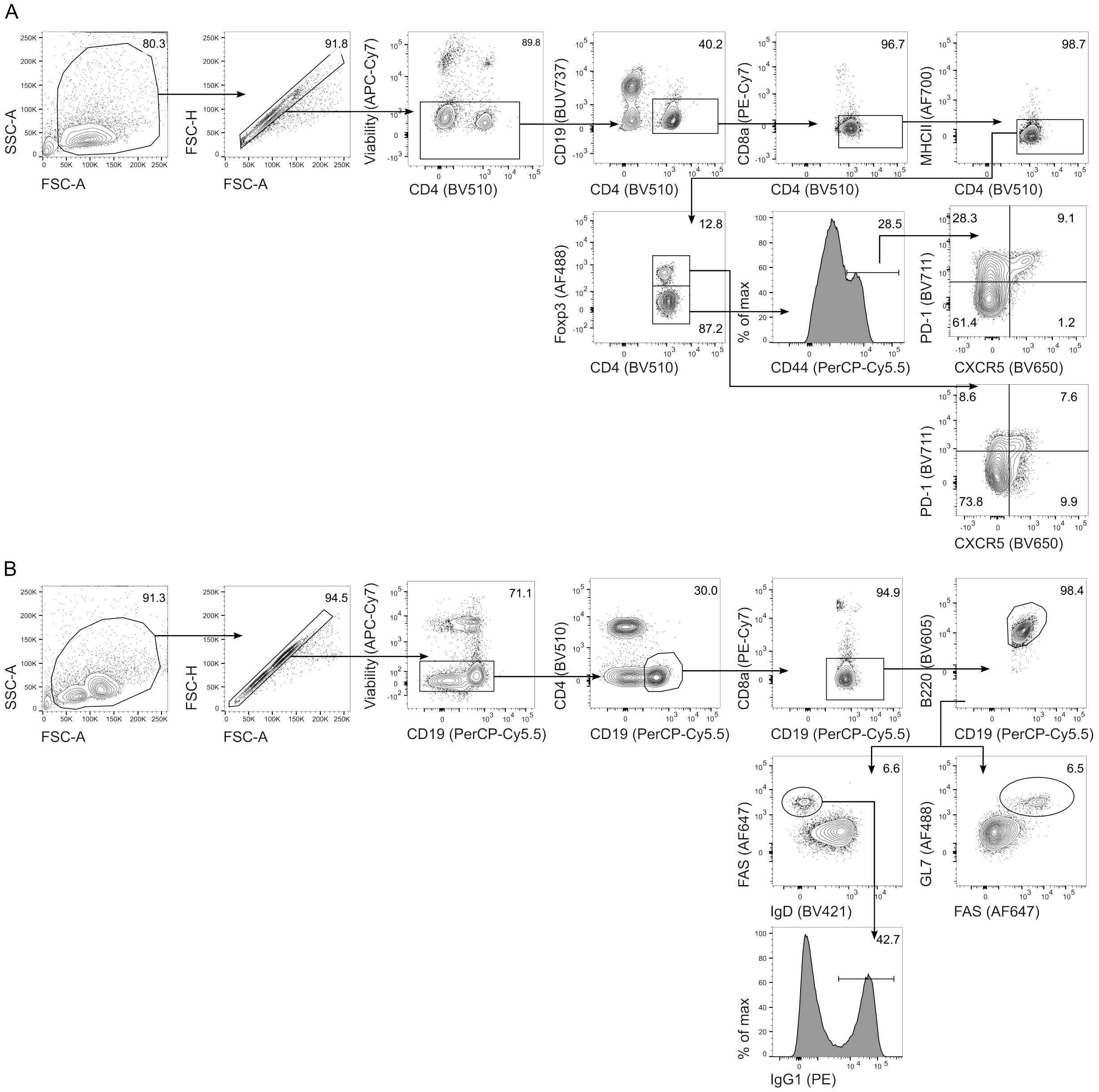

Figure S1: Gating strategy for the identification and analyses of cell populations by flow cytometry. (A) Gating strategy used in Fig. 1 to identify T_{FH} cells and to identify T_{FR} cells. **(B)** Gating strategy used in Fig. 2 to identify $FAS^{hi}IgD^{low}$ GC B cells, $FAS^{hi}GL7^{hi}$ GC B cells and in Fig. 3 to identify $IgG1^{+}$ GC B cells.

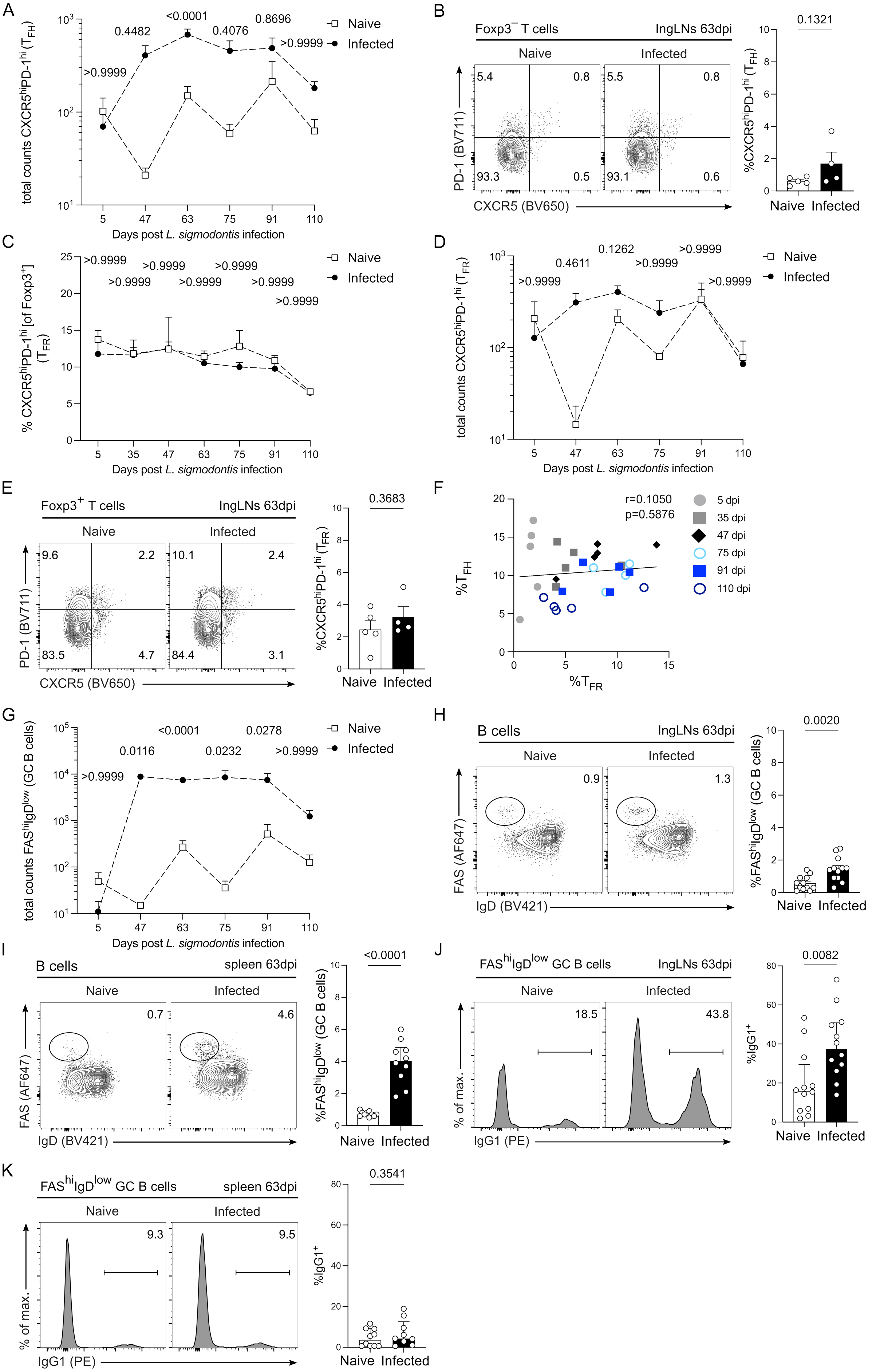

A
total counts CXCR5hiPD-1hi (TFH)
>0.9999
0.4482
<0.0001
0.4076
0.8696
>0.9999
Naive
Infected
Days post L. sigmodontis infection
B
Foxp3− T cells
IngLNs 63dpi
Naive
Infected
PD-1 (BV711)
CXCR5 (BV650)
5.4
0.8
93.3
0.5
5.5
0.8
93.1
0.6
0.1321
%CXCR5hiPD-1hi (TFH)
Naive
Infected
C
% CXCR5hiPD-1hi [of Foxp3+] (TFR)
>0.9999
Naive
Infected
Days post L. sigmodontis infection
D
total counts CXCR5hiPD-1hi (TFR)
>0.9999
0.4611
0.1262
>0.9999
>0.9999
>0.9999
Naive
Infected
Days post L. sigmodontis infection
E
Foxp3+ T cells
IngLNs 63dpi
Naive
Infected
PD-1 (BV711)
CXCR5 (BV650)
9.6
2.2
83.5
4.7
10.1
2.4
84.4
3.1
0.3683
%CXCR5hiPD-1hi (TFR)
Naive
Infected
F
r=0.1050
p=0.5876
5 dpi
35 dpi
47 dpi
75 dpi
91 dpi
110 dpi
%TFH
%TFR
G
total counts FAShiIgDlow (GC B cells)
>0.9999
0.0116
<0.0001
0.0232
0.0278
>0.9999
Naive
Infected
Days post L. sigmodontis infection
H
B cells
IngLNs 63dpi
Naive
Infected
0.9
1.3
FAS (AF647)
IgD (BV421)
0.0020
%FAShiIgDlow (GC B cells)
Naive
Infected
I
B cells
spleen 63dpi
Naive
Infected
0.7
4.6
FAS (AF647)
IgD (BV421)
<0.0001
%FAShiIgDlow (GC B cells)
Naive
Infected
J
FAShiIgDlow GC B cells
IngLNs 63dpi
Naive
Infected
18.5
43.8
% of max.
IgG1 (PE)
0.0082
%IgG1+
Naive
Infected
K
FAShiIgDlow GC B cells
spleen 63dpi
Naive
Infected
9.3
9.5
% of max.
IgG1 (PE)
0.3541
%IgG1+
Naive
Infected

Figure S2: T_{FH}, T_{FR} and GC B cell responses in *L. sigmodontis* infection. (A) Quantification of $CXCR5^{hi}PD\text{-}1^{hi}$ T_{FH} total cell counts in medLNs of naive and infected BALB/c mice. **(B)** Representative flow cytometry contour plots show the expression of CXCR5 and PD-1 among live $CD4^{+}CD8^{-}CD19^{-}Foxp3^{-}CD44^{+}$ T cells in ingLNs of naive and infected BALB/c mice at 63 dpi. Quantification of $CXCR5^{hi}PD\text{-}1^{hi}$ T_{FH} cells is displayed on the right. **(C)** Quantification of $CXCR5^{hi}PD\text{-}1^{hi}$ T_{FR} cell frequencies (of $Foxp3^{+}$ cells) in medLNs of naive and infected BALB/c mice. **(D)** Quantification of $CXCR5^{hi}PD\text{-}1^{hi}$ T_{FR} total cell counts in medLNs of naive and infected BALB/c mice. **(E)** Representative flow cytometry contour plots show the expression of CXCR5 and PD-1 among live $CD4^{+}CD8^{-}CD19^{-}Foxp3^{+}$ T cells in ingLNs of naive and infected BALB/c mice at 63 dpi. Quantification of $CXCR5^{hi}PD\text{-}1^{hi}$ T_{FR} cells is displayed on the right. **(F)** Correlation of $Foxp3^{+}CXCR5^{hi}PD\text{-}1^{hi}$ T_{FR} cell frequencies with $Foxp3^{-}CXCR5^{hi}PD\text{-}1^{hi}$ T_{FH} cell frequencies. **(G)** Quantification of $FAS^{hi}IgD^{low}$ GC B cell total cell counts in medLNs of naive and infected BALB/c mice. **(H)** Representative flow cytometry contour plots show the expression of FAS and IgD among live $CD19^{+}CD4^{-}CD8^{-}B220^{+}$ B cells in ingLNs of naive and infected BALB/c mice at 63 dpi. Quantification of GC B cells is displayed on the right. **(I)** Representative flow cytometry contour plots show the expression of FAS and IgD among live $CD19^{+}CD4^{-}CD8^{-}B220^{+}$ B cells in spleens of naive and infected BALB/c mice at 63 dpi. Quantification of GC B cells is displayed on the right. **(J)** Representative histograms show the expression of IgG1 on $CD19^{+}CD8^{-}CD4^{-}B220^{+}FAS^{hi}IgD^{low}$ GC B cells in ingLNs of naive and infected BALB/c mice at 63 dpi. Quantification of $IgG1^{+}$ GC B cells is displayed on the right. **(K)** Representative histograms show the expression of IgG1 on $CD19^{+}CD8^{-}CD4^{-}B220^{+}FAS^{hi}IgD^{low}$ GC B cells in spleens of naive and infected BALB/c mice at 63 dpi. Quantification of $IgG1^{+}$ GC B cells is displayed on the right. Each time point in (A, C, D, F) represents data from n=3-5 naive and n=4-14 infected BALB/c mice. Data from 5, 35, 47, 75, 91 and 110 dpi are from one experiment and data from 63 dpi consists of 2 independent experiments. Data in B and E represents data from n=5 and n=4 naive and infected BALB/c mice, respectively, from one experiment. Each time point in G represents n=3-19 naive and n=5-24 infected BALB/c mice. Data from 5, 47, 75, 91 and 110 dpi are from one experiment and data from 63 dpi consists of 2 independent experiments. Data in (H-K) represents n=10-12 naïve and n=9-12 infected BALB/c mice and were pooled from 2-3 independent experiments. Data was analyzed with either a two-way ANOVA with Bonferroni post-hoc test comparing naive and infected group differences per timepoint (A, C, D, G) or with a t-test (B, E, H, I, J, K) and shown as the mean ± SEM. Pearson correlation coefficient is stated in the plot (F). P-values are displayed as numerical values above the corresponding data points in the graphs.

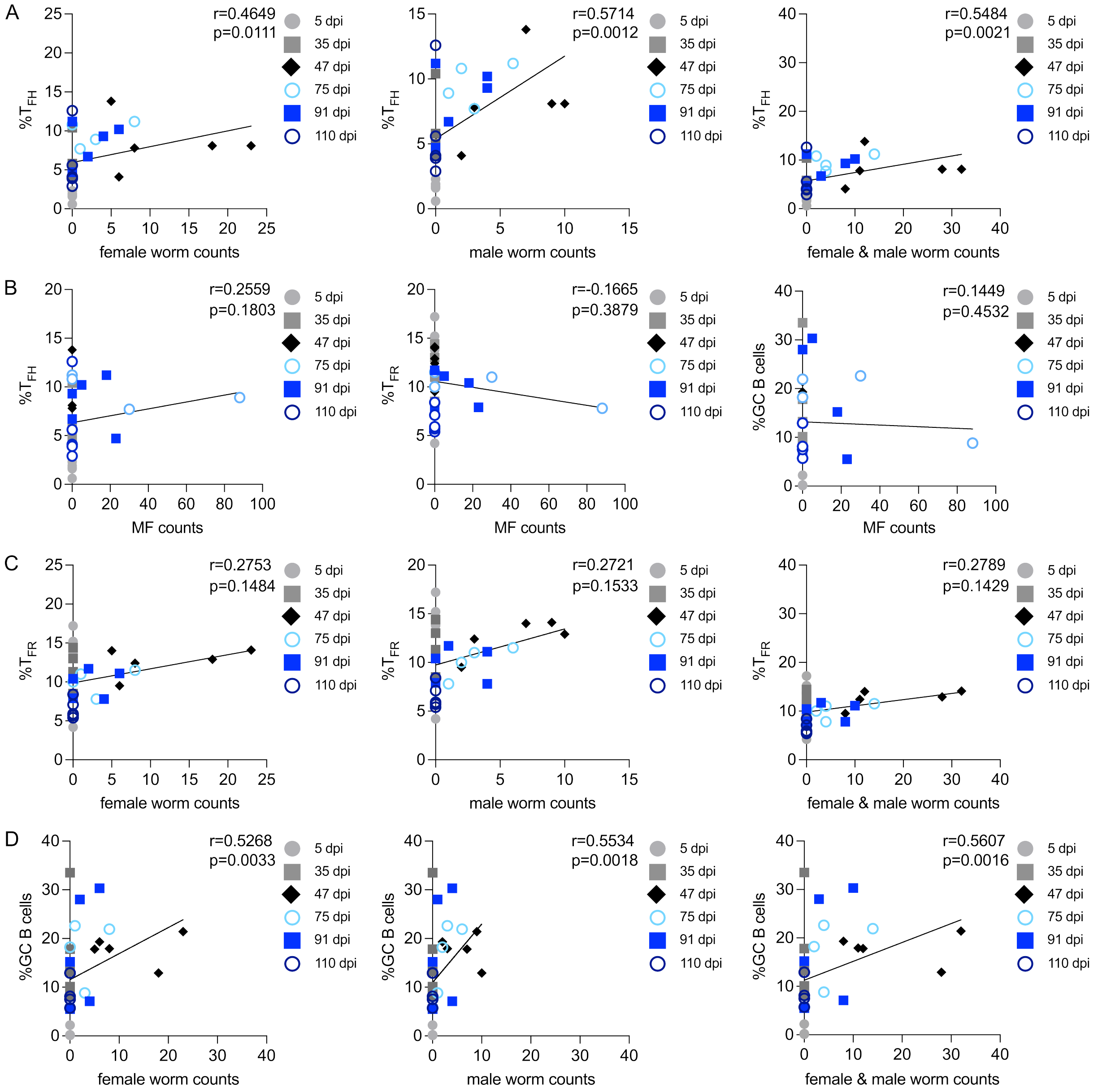

Figure S3: Correlation analyses of cell frequencies with adult worm counts and MF. (A) Correlation of Foxp3^{-}CD44^{+}CXCR5^{hi}PD-1^{hi} T_{FH} cell frequencies with female worm counts, male worm counts, and female and male worm counts. **(B)** Correlation of T_{FH} cell frequencies, T_{FR} cell frequencies and GC B cell frequencies with microfilariae (MF) counts. **(C)** Correlation of Foxp3^{+}CXCR5^{hi}PD-1^{hi} T_{FR} cell frequencies with female worm counts, male worm counts, and female and male worm counts. **(D)** Correlation of FAS^{hi}IgD^{low} GC B cell frequencies with female worm counts, male worm counts, and female and male worm counts. Spearman correlation coefficients and significances are stated in the plots.

Table S1: List of antibodies.

Marker	Fluorochrome	Source	Clone	Cat #	RRID
B220	BV605	Biolegend	RA2-6B2	103244	AB_2563312
CD4	BV510	Biolegend	RM4-5	100559	AB_2562608
CD8a	PE-Cy7	Biolegend	52-6.7	100722	AB_312761
CD16/32	-	Biolegend	93	101302	AB_312801
CD19	BUV737	eBioscience	eBio1D3	367-0193-82	AB_2895945
CD19	PerCP-Cy5.5	Biolegend	6D5	115534	AB_2072925
CD44	PerCP-Cy5.5	Biolegend	IM7	103032	AB_2076204
CXCR5	biotinylated	Biolegend	L138D7	145509	AB_2562125
FAS (CD95)	AF647	BD Biosciences	Jo2	563647	AB_2738346
Foxp3	AF488	eBioscience	FJK-16s	53-5773-82	AB_763537
GL7	AF488	eBioscience	GL-7	53-5902-82	AB_2016717
I-A/I-E	AF700	Biolegend	M5/114.152	107622	AB_493727
IgD	eF450	eBioscience	11-26c	48-5993-82	AB_1272202
IgG1	PE	Biolegend	RMG1-1	406608	AB_10551618
PD-1	BV711	Biolegend	29F.1A12	135231	AB_2566158
Streptavidin	BV650	Biolegend	-	405231	-
